# A BIOPSYCHOSOCIAL APPROACH TOWARD PREDICTING PHYSICAL ACTIVITY AMONG OLDER ADULTS WITH KNEE OSTEOARTHRITIS

**DOI:** 10.1093/geroni/igad104.2003

**Published:** 2023-12-21

**Authors:** Navin Kaushal, Donya Nemati, Thomas Best

**Affiliations:** Indiana University, Indianapolis, Indiana, United States; The Ohio state University, College of Nursing, Columbus, Ohio, United States; University of Miami, Miami, Florida, United States

## Abstract

Regular physical activity (PA) is imperative for preventing disability among older adults with knee osteoarthritis (KOA). However, PA is a complex behavior that relies on determinants from biological, psychological and social factors. Identifying determinants that predict PA would be informative for providing support for patients with KOA who are physically inactive. This study aimed to employ a biopsychosocial (BPS) approach to identify the strongest PA determinants among older adults with KOA.

Methods: Adults (age 65+) diagnosed with KOA (n=1,343) from the Osteoarthritis Initiative dataset were included to test the BPS model. Putative BPS determinants from baseline were identified by testing if they correlated with two-year follow-up change scores of accelerometry PA data. Each model factor was tested using ordinary least-squared regressions, and significant determinants were then used for the biopsychosocial model test, which employed hierarchical multiple regression.

Results: Bivariate correlations revealed 21 variables representing biological (n=13), psychological (n=4), and social (n=4) variables that correlated with change in levels of PA. Four significant variables were identified in separate regression models. Testing these predictors using hierarchical regression revealed the strongest determinant to be the 400-meter walk (β =-.26, p<.001), followed by age (β=-.23, p<.001), general health (β=-.15, p<.001), and BMI (β=-.21, p<.001).

Conclusion: The BPS model test revealed 50% of determinants to be mutable (fitness and BMI), and the remaining to be non/less mutable. The 400-meter walk test was the strongest indicator of future PA, suggesting that individuals with KOA who score low on this test could benefit from supported PA programs.

